# Functions of the Spinal Marrow

**Published:** 1856-07

**Authors:** 


*Functions of the Spinal Marrow.*—We take from the Southern Journal the following resumé of M. Brown-Séquard's experiments:

*M. Brown-Séquard's Discoveries of the Functions of the Spinal Marrow.*—Seldom has the scientific world been taken more by surprise than when M. Brown-Séquard announced his recent discoveries relative to the functions of the spinal marrow. Whatever may be wanting to complete our knowledge of the action of this portion of the nervous system, the brilliant investigations of Sir Charles Bell seemed to have set at rest forever the question as to the particular fibres which communicate motion to the muscles, and sensation to the brain. The theory of Bell, in a few words, is as follows: 'The spinal cord has two functions, relative to the two substances of which it is composed. It serves as an independent organ, detached from the brain, for the performance of reflex actions, a property which it owes to the grey matter contained in its centre. By the white substance it acts as a medium of communication between the brain and the parts to which the nerves are distributed, the posterior columns conveying sensations upward, and the anterior and lateral columns transmitting the power of motion in a downward direction. This theory was less the result of experiments upon living animals, than of a process of reasoning, Sir Charles having always manifested a strong repugnance to vivisections. M. Longet, however, demonstrated, by the application of galvanism to sections of the spinal marrow of animals, that irritation of the posterior columns caused no movement, while that of the anterior columns occasioned no pain. On the contrary, the galvanic current caused extreme pain when applied to the posterior columns above the transverse section of the medulla, and excited movements when directed through the anterior columns of the lower segment. The grey matter was found to be insensible to the irritation of electricity. The theory of Bell, so remarkable for its simplicity and apparently so perfectly supported by the demonstrations of one of the most eminent experimental physiologists, could not fail of universal adoption, and although pathological facts were occasionally made known which appeared to contradict, to some extent, its conclusions, it seemed natural to believe that these were inaccurately reported.

It will be observed, that in the experiments of M. Longet, the spinal cord was always completely cut across. We may not unreasonably ask whether the organ thus divided is in the same condition for transmitting sensation and the power of motion, as when its continuity is in a great part preserved, and why this method of experimenting was employed, instead of cutting through each portion in succession, and observing the effect produced upon the function attributed to that part? In reply to the latter inquiry, M. Longet states that the operation of laying bare the spinal marrow, and evacuating the fluid which is contained in the cavity of the arachnoid, is always followed by paralysis, both of sensation and motion, of the posterior extremities, thereby rendering further investigation impossible. Here was the great obstacle to researches in the functions of the spinal cord, and the removal of this obstacle was the first step taken by M. Brown-Séquard. He ascertained that the nervous disturbance following the opening of the spinal canal was caused by the loss of blood and by the pain and shock consequent upon the operation. By operating in such a manner as to prevent a great flow of blood, and by allowing the animal time to recover from the depressing effects of the operation, he found that both sensation and motion returned to the posterior extremities in almost, if not quite, their original degree.

Thus enabled to experiment upon the cord in a normal state (as far as its functions were concerned,) he proceeded to isolate various portions of the different columns by sections made with extreme care, and demonstrated a series of laws relative to the spinal functions, the principal of which are the following:

1. The posterior columns may be divided without destruction either of sensation or motion.

2. Sensation and motion are destroyed when the grey substance is cut across.

3. Integrity of the antero-lateral columns does not interfere with the loss of motion, nor does integrity of the posterior columns prevent loss of sensation.

4. Division of the posterior fibres of the cord, so far from abolishing sensation in the parts to which these fibres are distributed, appears, on the contrary, greatly to increase it.

5. When the posterior columns are divided, sensation continues to be transmitted between the lower portion and the grey substance, which transmits the impression to the sensorium by means of fibres descending from the upper portion, and joining obliquely the grey substance below the point where the section is made.

Our limits forbid us to detail the experiments upon which the above conclusions are founded. They have been repeated over and over again with the same results, in the presence of a committee appointed by the *Société de Biologie*, consisting of MM. Claude Bernard, Bouley, Brock, Giraldès, Goubaux and Vulpian, to whom was referred M. Brown-Séquard's memoir, and who were entirely satisfied with his conclusions. The interesting report which they made to the society, is the most convincing evidence of M. Brown-Séquard's skill as an experimenter and his eminence as a physiologist.—*Southern Journal.*

Dr. Bennett Dowler, of New Orleans, puts in a claim to originality in this discovery. We quote from the March number of the New Orleans Journal the more essential portion of his article:

"It is now more than a dozen of years since the writer of these lines began to publish his experimental researches and inquiries concerning this system. Hundreds of human beings were submitted to experiment immediately after death; many vivisections of animals were performed before numerous medical witnesses competent to judge of their validity and fairness—witnesses who agreed in their testimony, and had no rational motives to deceive. Animals were decapitated, their spinal cords divided transversely and longitudinally, without destroying sensation and voluntary motion; the posterior and anterior roots of the spinal cord were irritated, divided, and destroyed; the nerves were followed to their distributions, and dissected away, as the readers of this journal and other journals know, showing that all of the fundamental principles of Bell and Magendie, concerning the *two* sets, as well as Hall's *four* sets of nerves, were erroneous,—that the muscles possess an inherent power of contraction; that both the posterior and anterior nerve-roots give indifferently the signs of motion and sensation; that the twitching motions described by Bell and Magendie as exclusively pertaining to the anterior root, can always, contrary to their theory, be more fully produced after separation from the cord than before, by following the nerve toward its distribution; that the spinal cord can be divided in three or four places, and that the head may be removed without destroying sensation and voluntary motion, and that sometimes, in the alligator at least, the head on the one hand, and the separated body on the other, act in a voluntary manner each for itself, giving all the rational indications that could be expected of an animal, as purpose, feeling, &c.

"It is, however, in the decapitated body that all the conceivable phenomena characterizing sensation and voluntary motion are the most strikingly manifested, often for hours, with the exception of such phenomena as appertain exclusively to the special senses, as hearing, seeing, &c., the body acting of course like a blind animal until informed by touch."

